# Analysis of the approach angle to medial orbitotomy that avoids accidental neurotrauma in the mesaticephalic dog skull utilizing 3D computer models and virtual surgical planning

**DOI:** 10.3389/fvets.2023.1185454

**Published:** 2023-05-11

**Authors:** Michael C. Congiusta, Jason W. Soukup

**Affiliations:** Dentistry and Oromaxillofacial Surgery, Department of Surgical Sciences, School of Veterinary Medicine, University of Wisconsin-Madison, Madison, WI, United States

**Keywords:** medial orbitotomy, CT, 3D computer model, virtual surgical planning, approach angle

## Abstract

This study was conducted to determine an approach angle to medial orbitotomy that avoids accidental neurotrauma in mesaticephalic dogs. Medical records of dogs with mesaticephalic skulls that were presented to the veterinary medical teaching hospital for head computed tomography (CT) between September 2021 and February 2022 were reviewed. Descriptive data were queried, and CT findings were analyzed. Dogs greater than 20 kg and possessing a disease-free orbitozygomaticomaxillary complex (OZMC) on at least one side of the skull were included in this study. Digital imaging and communications in medicine (DICOM) files of head CT studies were imported into medical modeling software, and the safe approach angle for medial orbitotomy was determined using three-dimensional (3D) computer models and virtual surgical planning (VSP) principles. Angles were measured along the ventral orbital crest (VOC) from the rostral cranial fossa (RCF) to the rostral alar foramen (RAF). The safe approach angle at four points from rostral to caudal along the VOC was measured. The results at each location were reported as mean, median, 95% CI, interquartile ranges, and distribution. The results were statistically different at each location and generally increased from rostral to caudal. The variances between subjects and the differences between locations were large enough to suggest a standard safe approach angle in mesaticephalic dogs cannot be determined and should be measured for each patient. A standardized approach angle to medial orbitotomy is not possible in the mesaticephalic dog. Computer modeling and VSP principles should be implemented as part of the surgical planning process to accurately measure the safe approach angle along the VOC.

## Introduction

Orbitotomies are often needed in cases of larger, infiltrative malignant neoplasms, especially those involving the orbitozygomaticomaxillary complex (OZMC) (1–8). Orbitotomy techniques have been described in humans and the veterinary literature (1–6, 9). However, improvisation of surgical technique (e.g., angle adjustments and wider surgical margins) may be required to achieve maximal exposure while avoiding critical anatomical structures (10). Challenges encountered include complex maxillofacial anatomy, intraoperative complications risks, functional and esthetic considerations, and client expectations (11, 12).

Complications related to surgeries near the cranial vault can be devastating for the patient and may result in neurological deficits (13). Accidental penetration into the cranium can lead to life-threatening and severe post-operative complications, such as but not limited to, subdural hematoma development, blindness, seizures, raised intracranial pressures, and electrolyte imbalances (14, 15).

It is of paramount importance for the surgeon to avoid accidental penetration into the cranial vault. Establishing a safe approach angle for medial orbitotomy will improve preoperative planning and intraoperative guidance and may result in improved surgical outcomes. It has been suggested that the approach angle that avoids accidental penetration into the cranial vault is approximately 40° (15). However, a larger study investigating this angle and how it may change from rostral to caudal is warranted.

Computed tomography (CT) is widely used to assess the extent of infiltrative disease processes, demarcate between soft tissue and bone, and determine intended surgical margins (12, 16). Despite its limited contrast resolution, CT is the preferred imaging modality for 3D computer modeling and virtual surgical planning (VSP), owing to features of high spatial resolution and volumetric representation, particularly when considering osseous structures (12, 16–19). Digital imaging and communications in medicine (DICOM) files can be imported into software to create three-dimensional (3D) computer models (19). The operator can then generate 3D computer models +/- 3D-printed models for VSP (18, 19). Surgical planning using 3D models enhances the understanding of anatomical landmarks and pathology, improves surgical margin acquisition, and reduces intraoperative hemorrhage and surgical and anesthesia time (16, 20–22). The objective of this study was to investigate an approach angle that will enable the surgeon to avoid entering the cranial vault when performing a medial orbitotomy (19). We hypothesize that the safe approach angle to medial orbitotomy will vary minimally between individual mesaticephalic dogs and between horizontal positions along the medial orbit.

## Materials and methods

**Figure 1:**
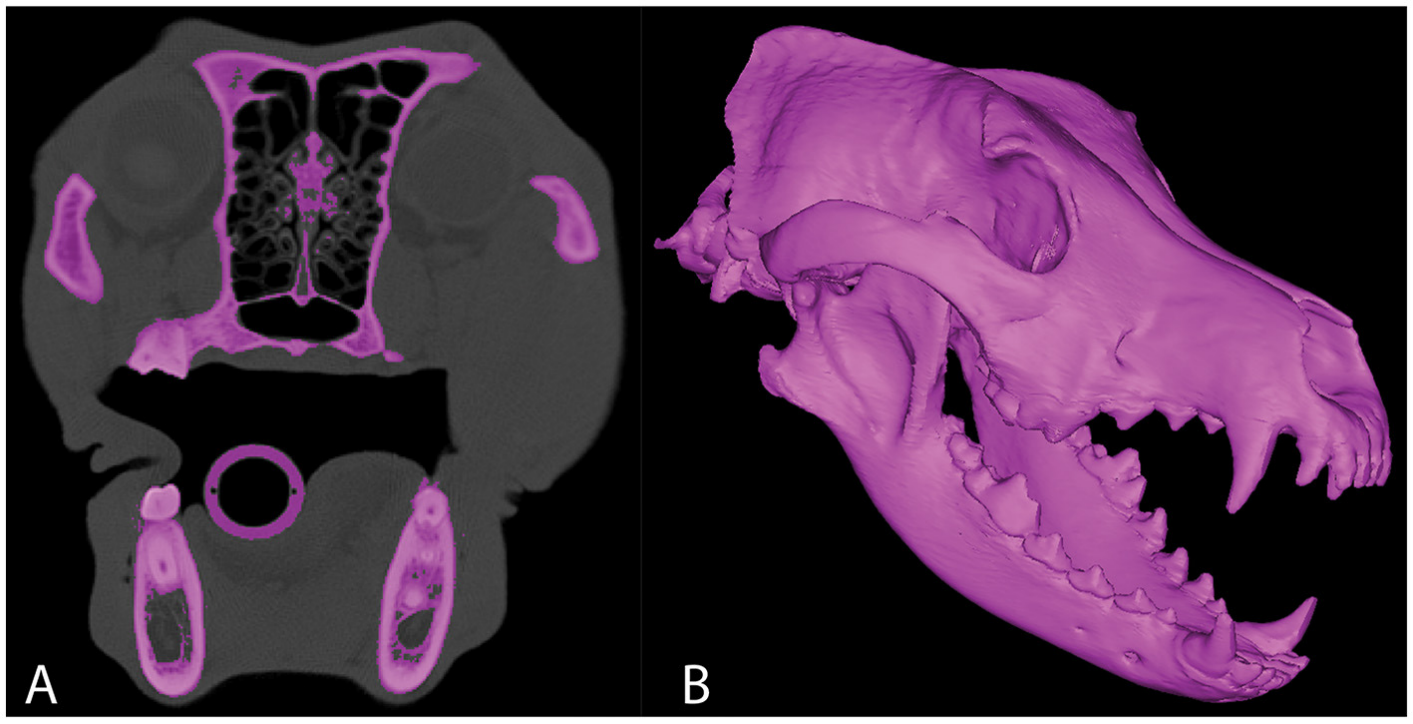
Representative axial CT image **(A)** in which segmentation of the skull has been performed. 3D computer model of the same CT study **(B)**.

**Figure 2:**
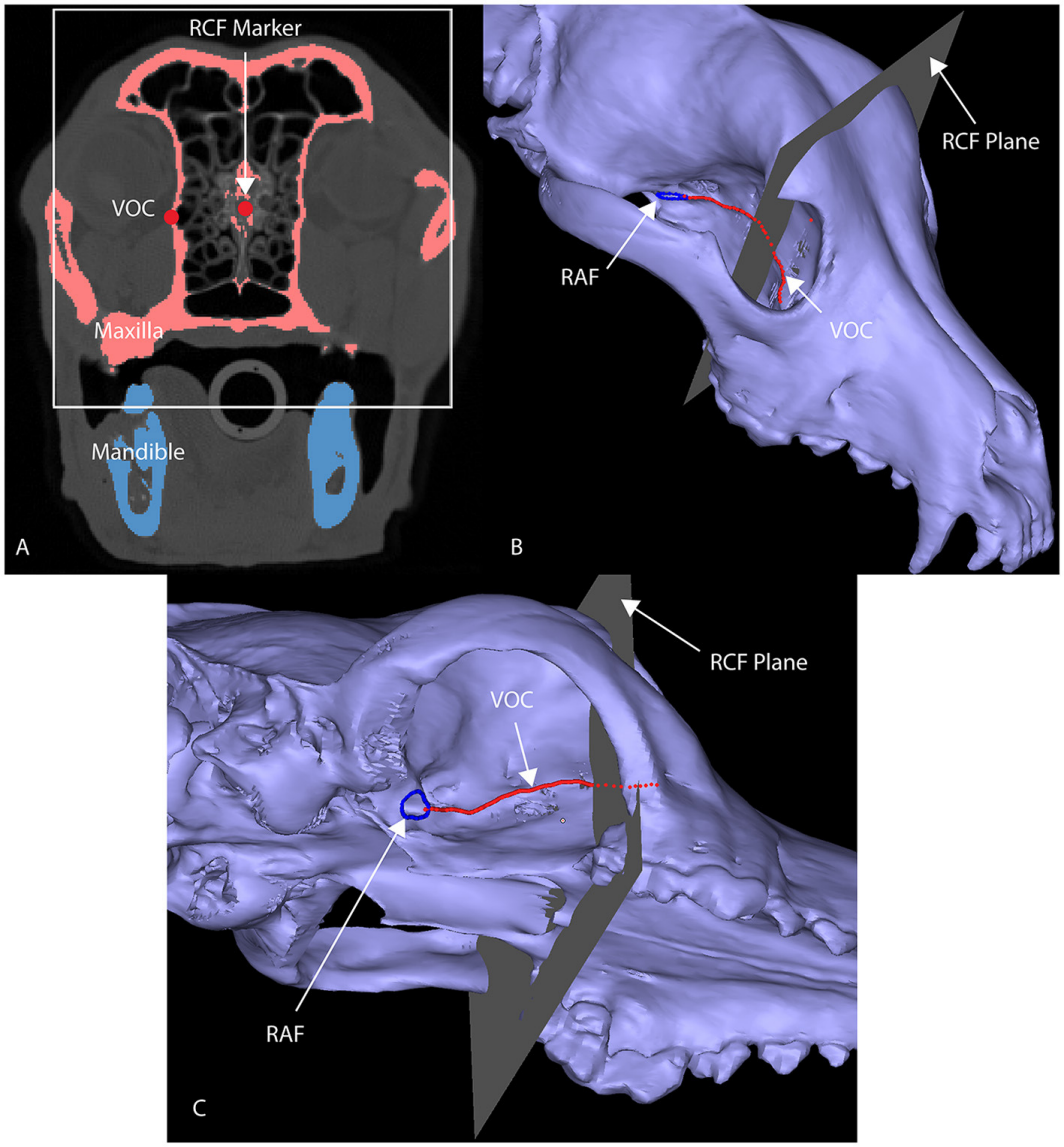
Axial CT image **(A)** of a segmented skull with a marker at the junction of the RCF and the cribriform plate **(A)**, designated as RCF marker. This marks the rostral origin of the VOC spline. A 3D computer model of a maxilla **(B, C)** highlights the anatomical markers utilized to develop the VOC spline (red line): the RAF (blue circle) and the intersection of the RCF and the cribriform plate [the gray plane in **(B, C)**; white box in **(A)**] have been delineated.

**Figure 3:**
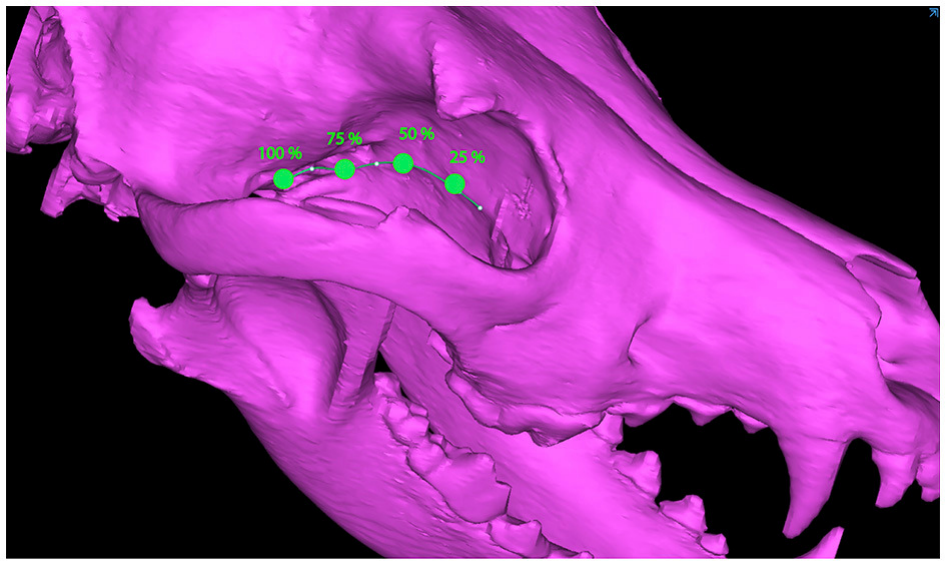
Subject skull depicting the 25, 50, 75, and 100% locations along the VOC spline.

Dogs with mesaticephalic skull types, weighing >20 kg with a head CT evaluated by the Dentistry and Oromaxillofacial Surgery Service at The University of Wisconsin-Madison Veterinary Medical Center between 8 September 2021 and 9 February 2022, were included in the study. The cephalic index (skull width/length x 100) was used to define the mesaticephalic skull shape and measured on a three-dimensional modeling software (Mimics 21.0, Materialize, Leuven, Belgium) (23). Skull width was defined as the maximum distance between opposing zygomatic arches, and skull length was defined as the distance from the occiput to the prosthion (23). Dogs with a cephalic index of +/-15% of the reported mesaticephalic average (56.0) were included in the study (23). The CT scan of each dog enrolled in the study required the side of the maxilla, in which measurements were to be taken to be free of pathology. Each subject was scanned by a 16-slice CT scanner at a slice thickness of 0.625 mm (GE Lightspeed, GE Healthcare, Milwaukee, WI). DICOMs were imported into a dedicated image segmentation and three-dimensional modeling software (Mimics 21.0, Materialize, Leuven, Belgium). A mask of the skull was created using a thresholding operation, and a 3D model of the subject skull was created as previously described (18, 19) (Figure 1). Within the 2D axial window, the intersection of the rostral cranial fossa (RCF) of the cranial vault and the cribriform plate was identified, and the geometric center was marked (Figure 2). An axial plane was inserted orthogonally into the sagittal plane at this point on the 3D computer model, signifying the intersection between the RCF and the cribriform plate. Subsequently, a spline interpolation function was used to trace the ventral orbital crest (VOC) from the aforementioned plane (representing the intersection of the RCF and the cribriform plate) to the rostral alar foramen (RAF) on the 3D computer model (Figure 2). Using Mimics 21.0 (Materialize, Leuven, Belgium), the spline interpolation function can be utilized to locate a curve that connects data points on the surface of the 3D computer model. This process provides an accurate method to record measurement points along the surface of a complex anatomical model. This spline interpolation function was used to delineate and define the curved VOC. The VOC was chosen as the most relevant and identifiable surgical landmark of the medial orbit. The spline length was recorded and subdivided into four isometric segments as follows: 25, 50, 75, and 100% of the total spline length (Figure 3). At each length, a marker was placed on the 3D computer model, which was also visible in the 2D CT windows.

**Figure 4:**
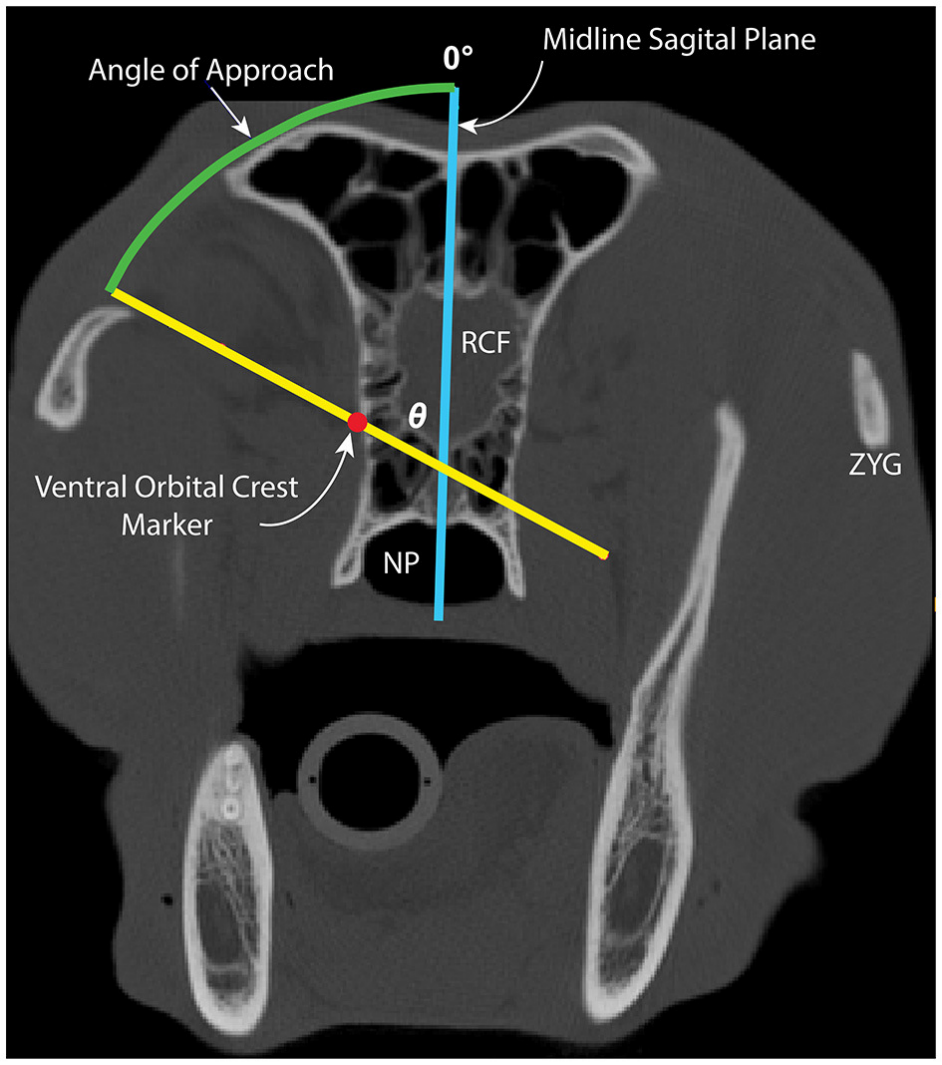
Image depicting measurement of the safe approach angle (*θ*; green arc) to medial orbitotomy at the 25% location. The midline sagittal plane (blue line) and line through the VOC marker, staying 1 mm away from the cranial vault (yellow line), are depicted. The midline sagittal plane represents *θ* = 0°. NP, nasopharynx; RCF, rostral cranial fossa; ZYG, zygomatic arch.

Within the 2D axial window, the 25% marker was located, and two lines were inserted as follows: (1) a vertical line along the midline sagittal plane and (2) a line extending through the 25% marker and intersecting with the midline sagittal plane staying 1 mm away from the cranial vault (Figure 4). The midline sagittal plane was designated as 0° (Figure 4). The angle between the VOC marker and the midline sagittal plane represents the safe approach angle (*θ*). This angle was obtained by consensus and recorded for each subject (Figure 4). This process was repeated at the 50%, 75%, and 100% locations for each subject.

### Statistical analysis

Descriptive statistics of the subjects are presented as mean (± SD), median, and ranges. The Shapiro–Wilk and Kolmogorov–Smirnov normality tests, which were performed at each location to confirm the approach angle data, were normally distributed. The means and associated 95% CIs as well as median, range, and interquartile ranges were computed for all the locations. A mixed effects ANOVA with location as a fixed effect and subject as a random variable was run to compare the approach angle between locations. If the ANOVA *p*-value was significant (*p* < 0.05), two-way *post-hoc* analyses with Tukey's family-wise correction were used. All analyses were performed in Prism 9.4.1 (GraphPad, San Diego, CA).

## Results

**Figure 5:**
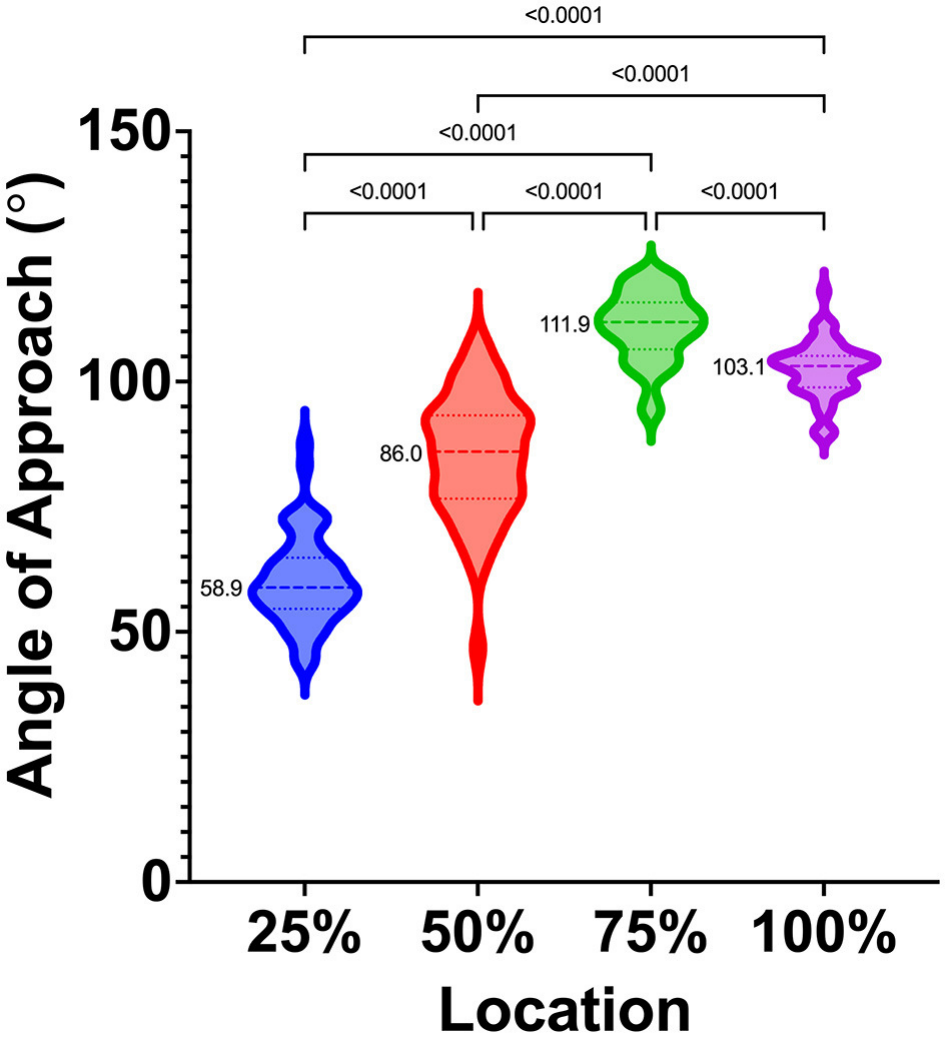
Violin plot depicting median (dashed lines), interquartile ranges (dotted lines), and distribution of safe approach angles at each location, from rostral (25%) to caudal (100%), along the VOC. Medians are also reported to the left of each violin plot. Significant differences between locations are indicated with solid bars and *p*-values.

A total of 48 dogs met the inclusion criteria and were included in the study. Subjects included in the study were one intact female dog (2.1%), eight intact male dogs (16.7%), 26 castrated male dogs (54.2%), and 13 spayed female dogs (27.1%). The group had a mean ± SD age of 7.5 ± 3.7 years and a median age of 7.2 years (range, 1.1–16.0 years). The mean ± SD body weight was 33.5 ± 9.9 kg, and the median (range) body weight was 32.2 kg (20.4–58.0 kg). The mean body condition score was 5.7 ± 1.2, and the median body condition score was 5/9 (range, 4/9 to 9/9). The following breeds were included: Labrador Retriever (n = 11), German Shepherd (8), German Shorthaired Pointer (5), Golden Retriever (5), Great Pyrenees (5), Rhodesian Ridgeback (1), Australian Shepherd (1), English Springer Spaniel (1), Goldendoodle (2), English Pointer (2), Black Mouth Cur (1), Mountain Feist (1), Beagle (1), Irish Setter (1), and Treeing Walker Coonhound (1); there were also two mixed-breed dogs [Pit Bull cross (1) and Labrador Retriever cross (1)]. The mean cephalic index was 54.9 ±3.4, and the median (range) cephalic index was 55.0 (48.4–62.5). The mean (95% CI) angles for the 25, 50, 75, and 100% locations were 60.3° (57.7–63.0°), 85.0° (81.5–88.6°), 111.0° (109.0–113.0°), and 102.5 (100.8–104.1°), respectively. The median (range) angles for the 25%, 50%, 75%, and 100% locations were 58.9° (43.9–87.7°), 86.0° (46.8–107.3°), 111.9° (93.99–121.3°), and 103.1° (89.4–118.0°), respectively. The results were statistically different at each location (*p* < 0.05), and median values, interquartile ranges, and distribution are presented (Figure 5).

## Discussion

Among the major outcome objectives, a medial orbitotomy is performed to avoid neurotrauma secondary to accidental penetration of the cranial vault and to minimize other less significant intraoperative and postoperative complications (e.g., hemorrhage, corneal abrasions, diplopia, ptosis, and globe malposition) (9, 11, 13, 24–32). A standardized approach angle could aid the surgeon in the attainment of aggressive wide resections without accidental entry into the cranial space and avoid subsequent iatrogenic brain injury.

Our results found that there is a significant variance in the safe approach angle between locations and between dogs. Therefore, the results do not support a standard approach angle to medial orbitotomies in mesaticephalic dogs and support the need for individualized preoperative surgical planning. Computer modeling and VSP principles were used to measure the safe approach angle to medial orbitotomy in this study, which has been shown to enhance preoperative surgical planning and improve working knowledge of normal and aberrant anatomy (12, 33–35). VSP and surgical simulation have increased surgeon confidence and ability to achieve tumor-free surgical margins (11, 12, 18, 33–35). In addition, VSP has decreased the knowledge gap for complex surgeries and made their performance more appealing (36–38). By using VSP principles, we were able to trace the VOC with precision and measure distances in all groups (25, 50, 75, and 100%) accurately. Division of the VOC into four groups (25, 50, 75, and 100%) allowed quantification of the influence of rostral–caudal location on the approach angle. While variation among locations and dogs was too high to recommend a standard approach angle, our results serve to provide guidance to surgeons. The safe approach angle generally increases from rostral to caudal.

The rostral cranial fossa, ventral orbital crest, and rostral alar foramen were the primary anatomical landmarks chosen to develop measurement locations. The point inserted at the RCF determined the most anterior extent of the brain. The point inserted at the RAF determined the most posterior extent of the feasibility of performing surgery without incurring trauma to the cranial vault. The VOC is the most easily identifiable intraoperative surgical landmark for medial orbitotomy. A 1-mm measurement inferior to the cranial vault at each measurement location (25, 50, 75, and 100%) was made as an additional margin of safety to help ensure that there would be no accidental penetration into the cranium. The inherent limitation of this safety margin did not consider the use of specific surgical instruments (i.e., osteotome with a surgical mallet), where mispositioning of the instrument may lead to detrimental consequences or induce crack propagation (39). Consideration should be given to the thickness of surgical instruments utilized, and thus, a greater safety margin may be needed (39). The piezosurgery surgical insert used by the authors has a thickness of 0.35 mm. Within the confines of the present study, the angle generated from the intersection of these points consistently bypassed the cranial vault with the safety margin utilized. Thus, the authors believe that the identification of these anatomical landmarks will enhance preoperative surgical planning and enable the surgeon to plan osteotomies without accidental penetration into the cranial vault (25, 28–30, 40, 41).

Cephalometrics was an important consideration for subject inclusion in the present study. We chose to study only mesaticephalic skull types based on the assumption that minimal variance would be present. The rostrum length and angle and the zygomatic arch width and depth of the neurocranium are similar among the mesaticephalic breeds (42). An average cephalic index for the mesaticephalic canine was previously reported as 56.0. A cephalic index range of +/-15% of this average was chosen to account for ambiguity and variability as to what is considered mesaticephalic in the literature. The cephalic index range of phylogenetically mesaticephalic skull types supports the cephalic index for the mesaticephalic range as a continuous variable (43). In the present study, the lowest cephalic index (48.4) and the highest cephalic index (62.5) were reported in an English Springer Spaniel and a Pit bull cross, respectively, which, based on their breed and cephalic index, were classified as having mesaticephalic skull types. While these breeds support the cephalic index as a continuous variable, there is ambiguity about classifying dog breeds within the literature. Future studies are needed to validate a classification system of skull types among dog breeds. Additionally, patients >20 kg were selected based on positive correlations between skull length/width and body weight (44, 45). However, it is possible that less variance would be present in individual dog breeds. Additionally, a standard safe approach angle may be present for brachycephalic or dolichocephalic skull types. Future studies to address these questions are warranted.

Surgical management of the infiltrative periorbital disease has led to the exploration of other minimally invasive surgical methods, such as the endoscopic dorsal sub-palpebral transconjunctival approaches (32). While these approaches prove to be generally safe and minimally invasive, several disadvantages were encountered by the authors, such as decreased width of the field of view and image resolution from endoscopic probes, the small working field observed within the orbital space, and the inability to assess hemorrhage or tissue debris through endoscopy (32). Adaptable techniques to enhance visualization intraoperatively and associated anatomical landmarks are essential for surgical efficiency, excellent exposure, and limited post-operative discomfort, as seen with the trans-frontal orbitotomy approach, simplified lateral orbitotomy approach, unilateral ventral transpalpebral anterior orbitotomy approach, and the intraoral and extraoral approach with transpalpebral exenteration (6, 10, 15, 26).

Virtual surgical planning (VSP) based on 3D models of subject skulls was utilized, and the measured angles were not evaluated with cadavers. Therefore, significant consideration was not given to anatomical barriers that may create challenges in achieving the safe approach angle. An obvious example would be the presence of the globe. Depending on the method used to achieve orbitotomy, the globe needs to be either retracted or removed. In many cases, the orbitotomy is being performed to surgically manage ocular or OZMC neoplasia. In such cases, the globe is typically excised and would not interfere with the safe approach angle to the medial orbit. In other cases, however, the globe would need to be retracted, perhaps to a significant degree. In the author's experience, the degree to which the globe must be retracted during a medial osteotomy is most significant when attempting to approach the caudal extent near the RAF, and the equipment utilized should be carefully considered to avoid damaging neighboring anatomy. A literature review neither finds any studies describing the safe limits of global retraction nor the consequences of excessive retraction in dogs.

Within the limitations of the study design, our results suggest that the safe approach angle to avoid accidental neurotrauma should be evaluated at various points along the vertical length of the osteotomy in individual patients before performing a medial orbitotomy. The use of 3D computer modeling and VSP is a useful tool for visualizing and measuring a safe approach angle to medial orbitotomy and should serve as an integral surgical planning component for complex surgeries involving the orbit.

## Data availability statement

The raw data supporting the conclusions of this article will be made available by the authors, without undue reservation.

## Author contributions

All authors of this work have made substantial contributions, the conception and design of the study, or acquisition of data, or analysis and interpretation of data, drafting the article or revising it critically for important intellectual content, and final approval of the version to be submitted.
