# Corrigendum: Analysis of the approach angle to medial orbitotomy that avoids accidental neurotrauma in the mesaticephalic dog skull utilizing 3D computer models and virtual surgical planning

**DOI:** 10.3389/fvets.2023.1271656

**Published:** 2023-09-29

**Authors:** Michael C. Congiusta, Jason W. Soukup

**Affiliations:** Dentistry and Oromaxillofacial Surgery, Department of Surgical Sciences, School of Veterinary Medicine, University of Wisconsin-Madison, Madison, WI, United States

**Keywords:** medial orbitotomy, CT, 3D computer model, virtual surgical planning, approach angle

In the published article, the reference for Pollard and Verstraete (2008) was incorrectly written as:

“Pollard RE, Verstraete FJM. The diagnostic yield of conventional radiographs and computed tomography in dogs and cats with maxillofacial trauma. *Vet Surg*. (2008) 37:294–9. doi: 10.1111/j.1532-950X.2008.00380.x”

It should be:

“Bar-am Y, Pollard RE, Verstraete FJM. The diagnostic yield of conventional radiographs and computed tomography in dogs and cats with maxillofacial trauma. *Vet Surg*. (2008) 37:294–9. doi: 10.1111/j.1532-950X.2008.00380.x”

The authors apologize for this error and state that this does not change the scientific conclusions of the article in any way. The original article has been updated.

